# Lipomatous Hypertrophy: An Accidental Finding in Heart

**DOI:** 10.1155/2019/8613724

**Published:** 2019-07-14

**Authors:** F. Petrelli, L. Spagnoli, S. Aversa, S. Tripodi, C. Bellan

**Affiliations:** ^1^Department of Medical Biotechnologies, University of Siena, Siena, Italy; ^2^Pathology Unit, Azienda Ospedaliera Universitaria Senese, Siena, Italy

## Abstract

Lipomatous hypertrophy is an uncommon benign lesion of the atrium, generally asymptomatic, characterized by unencapsulated accumulation of adipose tissue entrapping cardiomyocytes. This pathology generally remains unnoticed and often emerges as an occasional finding. Here, we report two cases from our hospital including a review of the available literature.

## 1. Introduction

Lipomatous hypertrophy is a benign cardiac mass characterized by an unencapsulated accumulation of mature adipose tissue within the interatrial septum, rarely in the atrium wall [1]. As most patients with this condition remain asymptomatic, the majority of cases are generally picked up as incidental findings at the time of cardiac imaging, surgery, or necropsy [2, 3]. Some symptomatic cases, however, are described in literature [4, 5].

Here, we report two cases of lipomatous hypertrophy referred to our hospital, one after surgical removal and the other at autopsy.

## 2. Case Report

The first case was found incidentally on preoperative investigation of a 73-year-old female patient with progressive coronary heart disease. The lesion, completely asymptomatic, was detected by transthoracic and transesophageal echocardiography and initially appeared as an exophytic mass occupying the right atrial cavity, lacking the typical hourglass morphology and appearing suspicious for atrial myxoma. Despite the absence of symptoms related to the lesion, the surgeons decided to remove it anyway in order to promote blood overflow through the atrial cavity and prevent later obstruction. The patient was subsequently admitted to the Cardiac Surgery Department for planned coronary artery bypass surgery and mass resection. During surgery, the lesion turned out to be also intraparietal but was totally resected. At macroscopic examination the lesion was 3 cm in diameter, yellowish, and slightly increased in consistency without a true capsule. Although surgery was successful, the patient died because of her illness.

The second case is a 73-year-old male patient with an incidental finding in autopsy of a lesion of 4 cm in diameter in the interatrial septum that appeared thickened. No clinical problems or suspicious symptoms had emerged from the clinical information gathered.

## 3. Results and Discussion

**Figure 1:**
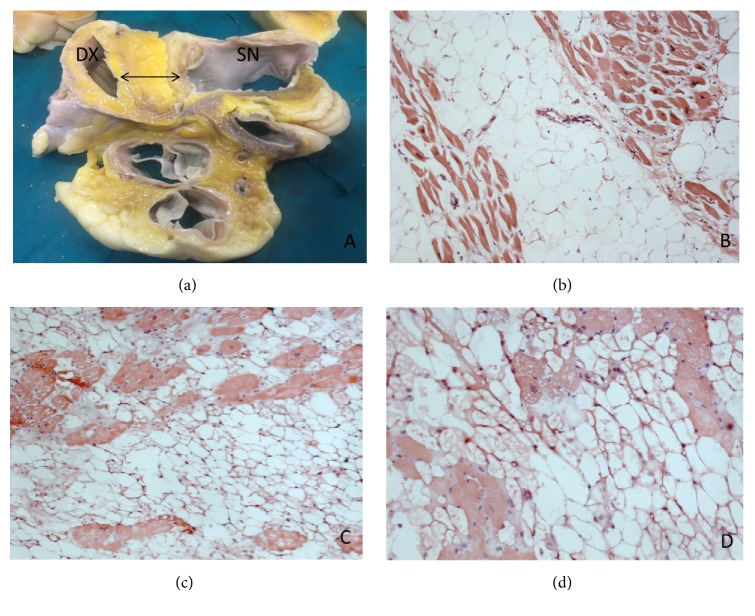
Macroscopic appearance of the lesion observed at autopsy (a) and histological overview showing extensive fat entrapping cardiomyocytes from atrial lesion of the first case (b) and from the interatrial septum of the second (c-d). Haematoxylin and Eosin 10X (b-c); Haematoxylin and Eosin 20X (d).

Both lesions, after fixation with 10% formalin solution, were sampled and embedded in paraffin to prepare 3-*μ*m-thick sections subsequently stained with haematoxylin-eosin. Histologically, both of the lesions demonstrated an unencapsulated accumulation of mature adipocytes with infiltrative growth pattern within hypertrophied cardiac myocytes. Lesion cells presented microvacuolated cytoplasm and centrally located nuclei, also exhibiting variation in shape and size, dispersed within the fat. Mitoses were absent (Figure 1). The pathologic differential diagnosis included cardiac myxoma, lipoma, and liposarcoma [6]. The diagnosis of myxoma was discarded due to the presence of fat and hypertrophied myocytes instead of the typical stellate or globular myxoma cells, characterized by abundant eosinophilic cytoplasm, indistinct cell borders, oval nuclei with open chromatin, and indistinct nucleoli, and of myxoid ground substance. Lipoma, unlike lipomatous hypertrophy, is an encapsulated lesion composed of only mature adipocytes and few, if any, myocytes. Finally, also well-differentiated liposarcoma could be excluded because it typically consists of lipoblasts containing large clear vacuoles and hyperchromatic indented nuclei [7].

Lipomatous hypertrophy represents a benign though rare condition that should always be considered in the differential diagnosis in case of an atrial mass detected during imaging, surgery, or necroscopy and definitively diagnosed by histological examination.
